# *Lactobacillus plantarum* in Dual-Species Biofilms With *Listeria monocytogenes* Enhanced the Anti-*Listeria* Activity of a Commercial Disinfectant Based on Hydrogen Peroxide and Peracetic Acid

**DOI:** 10.3389/fmicb.2021.631627

**Published:** 2021-07-30

**Authors:** Lourenço Bonneville, Vera Maia, Inês Barroso, Joaquín V. Martínez-Suárez, Luisa Brito

**Affiliations:** ^1^Linking Landscape, Environment, Agriculture and Food (LEAF), Departamento dos Recursos Naturais, Ambiente e Território (DRAT), Instituto Superior de Agronomia, University of Lisbon, Lisbon, Portugal; ^2^Departamento de Tecnología de Alimentos, Instituto Nacional de Investigación y Tecnología Agraria y Alimentaria (INIA-CSIC), Madrid, Spain

**Keywords:** *Listeria monocytogenes*, *Lactobacillus plantarum*, dual-species biofilms, refrigeration temperature, hydrogen peroxide/peracetic acid based disinfectant P3-Oxonia

## Abstract

The aim of this work was to investigate the effect of dual-species biofilms of *Listeria monocytogenes* with *Lactobacillus plantarum* on the anti-*Listeria* activity of a hydrogen peroxide/peracetic acid based commercial disinfectant (P3, Oxonia) when using conditions approaching the food industry environment. Nine strains of *L. monocytogenes*, including eight persistent strains collected from the meat industry and one laboratory control strain, were used in mono and in dual-species biofilms with a strain of *L. plantarum*. Biofilms were produced on stainless steel coupons (SSCs), at 11°C (low temperature) or at 25°C (control temperature), in TSB-YE (control rich medium) or in 1/10 diluted TSB-YE (mimicking the situation of biofilm formation after a deficient industrial cleaning procedure). The biofilm forming ability of the strains was evaluated by enumeration of viable cells, and the antibiofilm activity of P3 was assessed by the log reduction of viable cells on SSC. In both nutrient conditions and at low temperature, there was no significant difference (*p* > 0.05) between *L. monocytogenes* biofilm forming ability in mono- and in dual-species biofilms. In dual-species biofilms, *L. monocytogenes* was the dominant species. However, it was generally more susceptible to the lower concentration of P3 0.5% (v/v) than in pure culture biofilms. The presence of *L. plantarum*, although without significant interference in the number of viable cells of *L. monocytogenes*, enhanced the efficacy of the anti-*Listeria* activity of P3, since dual-species biofilms were easier to control. The results presented here reinforce the importance of the investigation into co-culture biofilms produced in food industry conditions, namely at low temperatures, when susceptibility to sanitizers is being assessed.

## Introduction

*Listeria monocytogenes* is an opportunistic foodborne pathogen responsible for listeriosis, a disease with high mortality rates (Buchanan et al., 2017). Listeriosis is mainly caused by the consumption of contaminated, predominantly ready-to-eat, food [European Food Safety Authority and European Centre for Disease Prevention and Control (EFSA and ECDC), 2021]. *Listeria monocytogenes* has been found in soil, water, plants, silage, sewage, slaughterhouse wastes, human, and animal feces and in a variety of foods (Farber and Peterkin, 1991). Decaying plant matter has been indicated as its natural niche (Vázquez-Boland et al., 2001). *Listeria monocytogenes* is a psychrotrophic bacterium that can survive and grow at refrigeration temperatures equal to 2°C (Gandhi and Chikindas, 2007). In the real scenario, *L. monocytogenes* tend to coexist with other species/genera forming biofilms at different temperatures, nutritional conditions, and surface types (Gilmartin et al., 2016). Therefore, in food processing environments, *L. monocytogenes* will most likely grow on surfaces with other microorganisms in mixed species biofilms (Carpentier and Chassaing, 2004; Habimana et al., 2009).

Lactic acid bacteria (LAB) can adapt and grow under different environmental conditions (Ringø and Gatesoupe, 1998; Castellano et al., 2004; McLeod et al., 2008). It is then likely that *L. monocytogenes* forms mixed species biofilms with LAB since numerous LAB have been isolated from food or the food industry environment (Carpentier and Chassaing, 2004; Habimana et al., 2009; Ait Ouali et al., 2014; Dutra et al., 2016). *Lactobacillus* spp. can be found in many different habitats including the surface of plants, soil, and the body of invertebrate and vertebrate animals (Duar et al., 2017). Many farm animals (i.e., swine and poultry) maintain dominant population of lactobacilli in their gut microbiota (Duar et al., 2017). Therefore, lactobacilli occur frequently in sewage from the meat processing industry as a result of fecal contamination (Duar et al., 2017).

Some authors reported that the interspecies interactions between *Staphylococcus aureus* and *Salmonella* had a negative effect on the antimicrobial resistance of each microorganism to cetrimonium bromide, peracetic acid, and sodium hypochlorite, compared with the monospecies biofilms (Iñiguez-Moreno et al., 2017). Other authors reported that dual-species biofilms of *L. monocytogenes* with *E. coli*, increased *L. monocytogenes* susceptibility to a combined enzyme-benzalkonium chloride (BAC) treatment (Rodríguez-López et al., 2017). This was not the case when the co-culture was with *Salmonella enterica*, which did not show any significant effect either on the biofilm-forming ability or on the antimicrobial resistance of *L. monocytogenes* (Kostaki et al., 2012). Contradictory results on the effect of LABs presence on the antibiofilm activity of disinfectants against *L. monocytogenes* have also been published. Some authors suggest that *L. monocytogenes* and *L. plantarum* dual species biofilms can be more resistant to disinfection treatments with BAC and peracetic acid than single species biofilms (van der Veen and Abee, 2011). Other authors claim that bacteriocin producing LAB helps to control *L. monocytogenes* biofilms (Guerrieri et al., 2009). Some LAB derived from kimchi (fermented vegetables) had also been reported as presenting inhibitory effects against *L. monocytogenes* biofilms on stainless steel surfaces (Hossain et al., 2020). Specifically, lactobacilli are potentially an antagonist of deteriorating and pathogenic bacteria due to the ability to produce a variety of inhibitory substances, such as organic acids, hydrogen peroxide, bacteriocins, and enzymes (Fijan, 2014; Ortiz et al., 2014a; Davoodabadi et al., 2015; Yeganeh et al., 2017; Abdelhamid et al., 2018; Fernandes, 2019; Vieco-Saiz et al., 2019).

The objective of this work was to evaluate the effect of *L. plantarum* in dual-species biofilms with *L. monocytogenes* on the anti-*Listeria* activity of a commercial disinfectant based on hydrogen peroxide/peracetic acid based commercial disinfectant (P3-Oxonia). Biofilms were produced in environmental conditions similar to those found in food processing plants after a poor cleaning procedure (abused refrigeration temperature – low temperature, stainless steel surface, and nutrient deprivation).

## Materials and Methods

### Bacterial Strains and Inocula Preparation

Nine strains of *L. monocytogenes* and one strain of *L. plantarum* were used in this study. The strains of *L. monocytogenes* were characterized as previously described (Table 1). Eight strains were persistently collected from the meat industry in Spain. The strains were chosen because they were previously fully characterized, some were whole genome sequenced, and represented different PFGE types and antimicrobial susceptibilities. Although the number of strains is limited, they are representative of the main subtypes of *L. monocytogenes* found in the meat processing environments. *Listeria monocytogenes* EGD-e was used as control/laboratory strain.

**Table 1:** Characterization of the strains.

Species	Strain	Source	References
*Listeria monocytogenes*	S1 (R)	Swine (pig abattoir and pork processing plants)	Ortiz et al., 2014b, 2016; López-Alonso et al., 2015
	S2-1		Ortiz et al., 2014b
	S1 (S)		Ortiz et al., 2014b, 2016
	S10-1		Ortiz et al., 2014b, 2016; López-Alonso et al., 2015
	S2-2		Ortiz et al., 2014b, 2016; López-Alonso et al., 2015
	R6	Broiler (retail)	López-Alonso et al., 2019, 2020
	A7	Broiler (abattoir)	López-Alonso et al., 2019, 2020
	P12	Broiler (processing plant)	López-Alonso et al., 2019, 2020
	EGD-e	Control	ATCC® BAA-679™ https://www.ncbi.nlm.nih.gov/bioproject/PRJNA276/
*Lactobacillus plantarum*	LB95	Fermented olives	Dutra et al., 2016

*Lactobacillus plantarum* strain LB95 was collected from the fermented olives of Portuguese cultivars. Multiple bacterial species were able to coexist and form biofilms in food processing plants. *Lactobacillus* was chosen as an example or as a model of non-pathogenic bacteria, which are common in foods and food processing environments. The mixed biofilms of *Listeria-Lactobacillus* were considered as a good model because it has been recognized to be more likely that one particular type of pathogen would form mixed biofilms with commensal or spoilage bacteria, which are more common in the environment (Wang, 2019).

Cultures were maintained on tryptic soy yeast extract broth (TSB-YE; Biokar Diagnostics, Beauvais, France) containing 15% (v/v) glycerol and stored at −80°C. *Listeria monocytogenes* strains from the −80°C stock collection were streaked onto tryptone soy yeast extract agar (TSA-YE) and grown overnight at 37°C. *Lactobacillus plantarum* was streaked onto Man-Rogosa-Sharpe (MRS; Biokar Diagnostics, Beauvais, France) and grown for 48 h at 37°C. Subsequently, isolated colonies were picked from the plates to prepare a work collection into the respective semi-solid medium [MRS was supplemented with CaCO_3_ (1 g L^−1^) in order to neutralize the acid produced and preserve the viability of the cultures (Kirsop and Snell, 1984)]. After incubation the work collection was kept at 4°C until use.

In order to prepare the inocula for producing pure culture biofilms, *L. monocytogenes* strains from the work collection were inoculated onto TSA-YE plates. After a 24 h period of incubation at 37°C, the inocula was prepared by suspending one isolated bacterial colony in 10 ml of TSB-YE or 1/10 diluted TSB-YE (approximately 10^7^ CFU ml^−1^ confirmed through plating). Similarly, *L. plantarum* was inoculated onto MRS agar and incubated for 48 h at 37°C. After this period, the procedure for inocula preparation was the same as for *L. monocytogenes* strains. The use of 1/10 diluted TSB-YE compared with the control rich medium (TSB-YE) intended to mimic the situation of biofilm formation after a deficient cleaning procedure in the food processing industry.

### Disinfectant Agent

P3-Oxonia active (P3; Ecolab, Saint Paul, Minnesota, United States) was used for the evaluation of anti-Listeria activity. The active compounds of P3 are hydrogen peroxide, acetic, and peracetic acid. According to European Standard EN 13697 (European Committee for Standardization, 2001), P3 was diluted using sterile hard water (magnesium chloride, calcium chloride, and sodium bicarbonate, pH 7 ± 0.2) to achieve the tested concentrations. Exposure to P3 was at room temperature (25°C) in three different conditions: 0.5% (v/v) for 5 min, 1% (v/v) for 10 min, and 2% (v/v) for 10 min.

### Biofilm Formation on Stainless Steel Coupons

The procedure for biofilm formation was as described by Costa et al. (2016), with some modifications. Briefly, biofilms were formed on stainless steel coupons (SSCs; 1 mm thick, 10 mm × 10 mm, type 304, 2B finish; Metalurgica Quinacorte, Lda, Lousa, Portugal) in P24 microplates (Orange Scientific, Braine-l’Alleud, Belgium) and sealed with parafilm (Bemis, United States). Before use, SSC were cleaned in acetone to remove grease, rinsed in distilled water, and immersed in a phosphoric-acid-based cleaner (CIP 200, Steris Corp., Mississagua, Ontario, Canada) at room temperature for 20 min, rinsed again in distilled water, and sterilized in test tubes by autoclaving for 15 min at 121°C.

### Mono-Species Biofilm of *Listeria monocytogenes*

A coupon was placed per well, and 1.5 ml of the respective inoculum was added. Controls with non-inoculated coupons were present in each P24 microplate. Incubation was performed at 25°C (room temperature) for 48 h or at 11°C (low temperature) for 7 days, in both cases without agitation. After this period, in order to remove planktonic cells, each coupon was rinsed through pippeting 1 ml of Ringer’s solution (Sigma–Aldrich, St. Louis, MO, United States) on each side of the coupon, which was then placed in a new P24 microplate that contained a layer of 20 sterile glass beads (*ø* = 3 mm). Thirty sterile glass beads and 1 ml of Ringer’s solution were then added to each well. The microplate was stirred in a Microplate vortex (Tittertek DSG, Flowlabs, Germany) for 1 min at maximum speed (position 10) in order to remove the biofilm from both sides of the coupons. From each well, 100 μl of the suspension was withdrawn in order to perform serial decimal dilutions to spot inoculate (25 μl) TSA-YE plates. Colony forming units (CFUs) were counted after overnight incubation at 37°C. The assays were replicated using four replicates: two biologically independent cultures on distinct days (two biological replicates), each with two repetitions under identical conditions (two technical replicates).

### Dual-Species Biofilms of *L. monocytogenes* With *L. plantarum*

The procedure was the same to the one described for *L. monocytogenes* pure culture biofilms, except that at day 1, only *L. plantarum* was inoculated (1 ml) into the wells with the coupons. After 24 h at 11°C, *L. monocytogenes* inoculum was added (1 ml) to the wells with *L. plantarum* in order to produce dual-species biofilms. This was needed since simultaneous co-inoculation resulted in complete absence of detectable cells of *L. plantarum* in the biofilms. The assays also included wells for pure culture *L. plantarum* biofilms in which, after 24 h, instead of *L. monocytogenes* inoculum, 1 ml of fresh TSB-YE was added. A similar procedure was performed when the biofilms were cultured in 1/10 diluted TSB-YE. Incubation proceeded at 11°C for 7 days without agitation.

After this period, biofilms were removed as described in the mono-species biofilm paragraph, except that suspensions from co-culture biofilms were additionally plated onto PALCAM (Biokar Diagnostics, Beauvais, France), in order to count *L. monocytogenes* CFU. Plates were incubated at 37°C for 48 h before counting the colonies. *Lactobacillus plantarum* was indirectly calculated by subtracting to the total CFU numbers in TSA-YE, the number of *L. monocytogenes* in PALCAM. Similarly to pure culture biofilms, two biological replicates with two technical replicates were always performed.

### Antibiofilm Activity of P3

After biofilm formation, each coupon was rinsed with 1 ml of Ringer’s solution on both sides, placed in a new P24 plate and exposed to 1 ml of P3 at the respective concentration (0.5, 1, or 2%) and for the respective exposure time (5 or 10 min). For calculating log reduction, a control exposed to sterile water was used. After the exposure period, each coupon was rinsed with 1 ml of Ringer’s solution, and then transferred to a new P24 with a 20-glass bead layer in the bottom. A set of 30 glass beads was put on the top of each coupon and 1 ml of Dey/Engley neutralizing broth solution (D/E; Difco Laboratories, New Jersey, United States) was added for an efficient neutralization contact period of 5 min. After this step, the plate was stirred in a Microplate vortex for 1 min at maximum speed. From each well, 100 μl was directly inoculated and the remaining suspension was decimal diluted for spread inoculating TSA-YE plates or TSA-YE and PALCAM plates, for *L. monocytogenes* biofilms cells or dual-species biofilm cells, respectively. The CFU count assessment was made after 24 h and confirmed after 48 h of incubation at 37°C. Two biological replicates were performed with two technical replicates, each.

The disinfectant treatment was considered effective when a 4-log CFU reduction was obtained (European Committee for Standardization, 2001). The reduction was obtained by calculating the difference between the counts (log CFU cm^−2^) on the surfaces (SSC) exposed to (i) hard water (enumeration of viable cells) and (ii) to the disinfectant (antibiofilm activity of P3). If that reduction was observed with the lower P3 concentration and time exposure, no more treatments were carried out. Otherwise, treatments with higher concentration and/or exposure time were performed until the 4-log reduction was reached.

### Data Analysis

Data from biofilm formation on SSC (log CFU cm^−2^) and antibiofilm activity of P3 (log reduction) assays was checked for agreement to the normal distribution and for homogeneity of variance (Anderson-Darling test and Levene’s test, respectively) by using the MiniTab17 software (Minitab, Inc., Pennsylvania, United States). When normality and homogeneity of variance were confirmed, one-way ANOVA with Tukey’s test was performed to calculate statistical differences between average values. When the data did not comply with ANOVA assumptions, the non-parametric Kruskal–Wallis median test was used. A principal component analysis (PCA) and a Cluster Analysis (CA) were performed in order to compare the susceptibility of the nine *L. monocytogenes* strains in mono- and in dual-species biofilms with *L. plantarum* following exposure to P3 0.5% (v/v) for 5 min (log reduction). Biofilms were produced in SSC, for 7 days at 11°C, and grown in TSB-YE (control rich medium) or in 1/10 diluted TSB-YE (mimicking the situation of biofilm formation after a deficient industrial cleaning procedure). The CA was made using the hierarchic clustering method and Euclidean distance was used as a measure of similarity or distance between strains. The software used was Statistica version 7.0 (Statsoft Inc., Tulsa, United States). For all tests, a probability greater than 95% (*p* < 0.05) was considered as significant.

## Results

### Antibiofilm Activity of P3 Against Pure Culture Biofilms of *L. monocytogenes*

**Figure 1:**
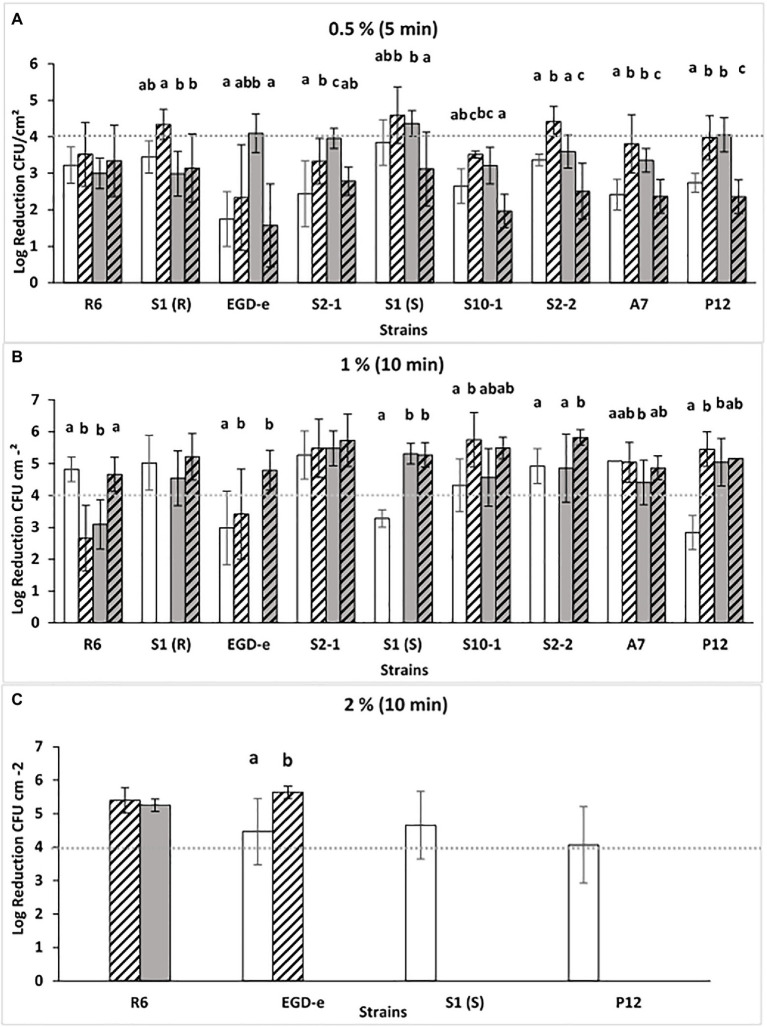
Log reductions (log CFU.cm^−2^) obtained after P3 treatment [0.5, 1, or 2% (v/v), A, B or C] for 5 or 10 min (**A-C**) of *Listeria monocytogenes* biofilms grown at different conditions: TSB-YE, 25°C (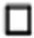), 1/10 TSB-YE, 25°C (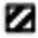), TSB-YE, 11°C (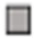), or 1/10 TSB-YE. 11°C (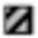). The disinfectant treatment was considered as effective when 4-log reduction in viable bacterial counts was reached. For each culture, different letters in the columns indicate significant differences (*p* < 0.05). At least two biological replicates were performed, with two technical replicates each.

Biofilms of *L. monocytogenes* were produced in SSC under four different conditions intended to mimic the situation of poor cleaning procedures in the food processing industry: In 1/10 diluted TSB-YE, compared with the rich control medium (TSB-YE); and at low temperature (11*°*C, for 7 days) compared with the ambient control temperature (25°C for 48 h). The different periods of incubation were previously calculated in order that biofilms produced at both temperatures presented similar levels of *L. monocytogenes* CFU. P3 antibiofilm activity was tested in rising concentrations [0.5, 1, and 2% (v/v)] of the disinfectant until the 4-log threshold was reached (Figure 1).

Compared with the other concentrations used [1 and 2% (v/v)], with the lower concentration of P3 [0.5% (v/v)], a general low susceptibility (<4-log CFU reduction) of the strains was observed (Figure 1). This effect was particularly noticed when biofilms were produced in conditions mimicking food industry environment (11°C in 1/10 diluted TSB-YE) (Figure 1A). The number of CFU in the biofilms did not affected these results, since cellular enumeration in SSC of biofilms of these strains grown at 11°C in TSB-YE and in 1/10 diluted TSB-YE were evaluated previously for these strains and showed no significant differences (*p* > 0.05; Barroso et al., 2019).

Since none of the nine isolates reached the 4-log reduction threshold in the four conditions tested, P3 concentration and the time of exposure were increased to 1 and 2% (v/v) for 10 min. With 1% (v/v) P3, all the strains, at least in one condition, reached the 4-log reduction threshold (Figure 1B). However, it was necessary to increase the P3 concentration to 2% (v/v) in order that all strains, in the four different conditions, were able to reach the 4-log reduction threshold (Figure 1C). The testing of increasing of the concentration is important to assess the concentration required to ensure the disinfectant efficacy (4-log CFU reduction threshold, European Standard EN 13697 2001; (European Committee for Standardization, 2001).

### Dual-Species Biofilms: Cellular Enumeration and P3 Antibiofilm Activity

As biofilms of *L. monocytogenes* in monoculture produced at low temperature were less susceptible to disinfectants, the assays with dual-species biofilms proceeded at 11°C, in 1/10 TSB-YE as well as in rich control medium TSB-YE. The results shown that in general, regardless the growth medium, *L. monocytogenes* was the dominant species in the dual-species biofilms with significantly higher numbers of viable cells (*p* < 0.05) (Supplementary Material). There were no significant differences (*p* > 0.05) between the average numbers of log CFU cm^−2^ of *Listeria* and the total biofilm population. Moreover, in both nutrient conditions, there was no significant difference (*p* > 0.05) between *L. monocytogenes* cell enumeration in mono- and in dual-species (Supplementary Material).

**Figure 2:**
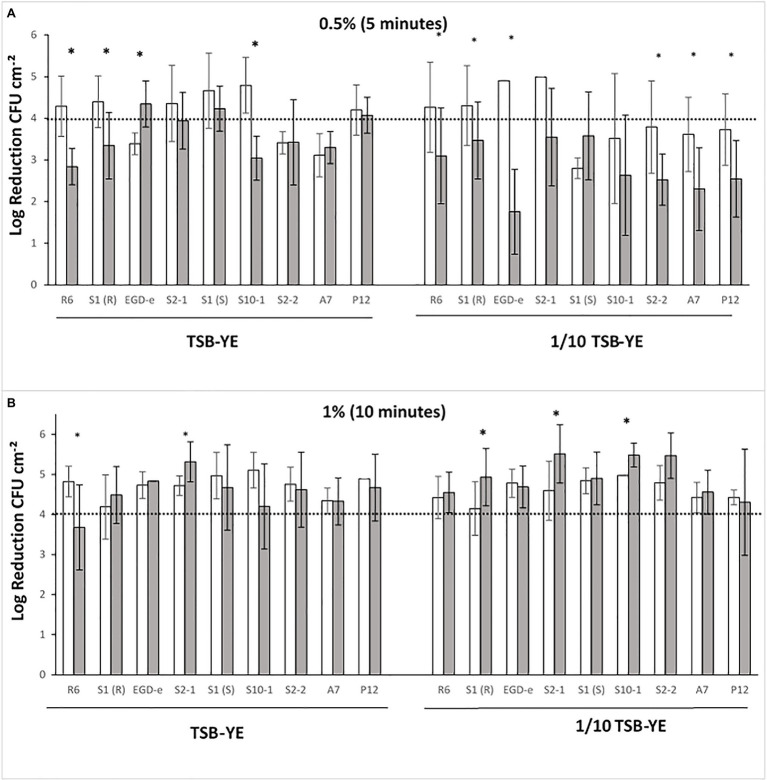
*L. monocytogenes* log reductions (log CFU cm^−2^) obtained after P3 treatments of mono- (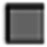) and dual-species biofilms with *Lactobacillus plantarum* (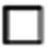) grown in rich control medium TSB-YE or in 1/10 diluted TSB-YE. **(A)** Biofilms exposed to 0.5% (v/v) P3, 5 min; **(B)** Biofilms exposed to 1% (v/v) P3, 10 min. The disinfectant treatment was considered as effective when 4-log reduction in viable bacterial counts was reached. For each culture, (*) in the columns indicate significant differences (*p* < 0.05). At least two biological replicates were performed with two technical replicates each.

Biofilms were exposed to P3 (Figure 2) and the anti-*Listeria* activity of the disinfectant was compared between mono- and dual-species biofilms with *L. plantarum*. LAB was never detected after the exposure of biofilms to any concentration of P3 tested suggesting that the eventual survivors would be outside the detection limit of the method. When grown in TSB-YE and exposed to 0.5% (v/v) P3, from the nine tested strains, six in mono-species [R6, S1(R), S2-1, S10-1, S2-2 and A7] were below the 4-log reduction threshold (Figure 2A). This effect was more noteworthy when biofilms were grown in 1/10 diluted TSB-YE, where the only four strains that reached the 4-log reduction threshold [R6, S1(R), EGD-e and S2-1] were in dual-species with *L. plantarum* (Figure 2A). In this scenario, six strains [R6, S1(R), EGD-e, S2-2, A7 and P12] out of the nine tested showed significant increased susceptibility to P3 when in dual-species biofilms with *L. plantarum* (*p* < 0.05) (Figure 2A). The concentration and the time exposure of P3 were increased to 1% (v/v) for 10 min in order to reach the 4-log threshold (Figure 2B). In these conditions, the majority of the strains in mono- and dual-species biofilms reached the reference level of reduction, regardless the biofilm growth medium (Figure 2B).

### PCA of Biofilm Susceptibility to P3

**Figure 3:**
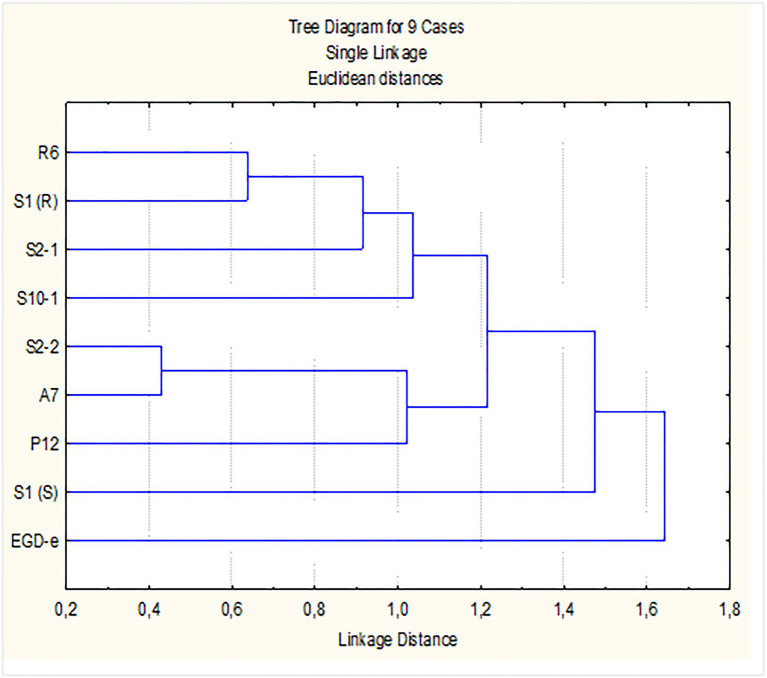
Dendrogram for *L. monocytogenes* strains. Dendrogram was defined by P3 [0.5% (v/v), 5 min] log reduction values (susceptibility) of biofilms, in mono- and in dual-species with *Lactobacillus plantarum*, produced in SSC, for 7 days at refrigeration temperature (11°C), in control rich medium (TSB-YE) and in 1/10 diluted TSB-YE (mimicking poor cleaning procedures).

Accordingly, the differentiation of *L. monocytogenes* strains based on their response to the lower concentration of P3 tested [0.5% (v/v)] was investigated. Data from log-reduction of mono and-dual species biofilms grown in both nutrient media, TSB-YE and 1/10 TSB-YE, at 11°C, were used for PCA. The initial four-dimensional space (four variables/conditions) were reduced to a plane F1F2 defined by the two first principal components. The projection of the nine strains in this plane, which accounted for 70.4% of the variability of the data, showed that the eight persistent strains were gathered in a major cluster. Strains S2-1, S1(S), S1(R), R6, and S10-1 with higher log reduction values when in mono species grown in 1/10 TSB-YE and in dual-species in TSB-YE; and strains P12, S2-2, and A7 with lower log reduction values in the same conditions. Strain EGD-e, although with lower log reduction values when in mono species grown in 1/10 TSB-YE and in dual-species in TSB-YE was well separated from the eight persistent strains (data not shown). Cluster analysis (Figure 3) confirmed the presence of the two clusters of strains suggested by PCA. In fact, when a linkage distance of about 1.5 is used, the nine *L. monocytogenes* strains could be grouped in two clusters: the control strain EGD-e in one cluster and the eight industrial persistent strains in the other cluster (Figure 3).

## Discussion

### Pure Culture Biofilms of *L. monocytogenes*

*Listeria monocytogenes* is resistant to stress conditions encountered in food processing environments, making control of this organism in food production and processing environments difficult. It is therefore important to understand how the food industry environment dictates the biofilm forming ability and the biofilm susceptibility of *L. monocytogenes*. Biofilm formation assays on SSC showed that neither different nutrient availability nor different temperatures had significant effect on biofilm formation.

By contrast, the antibiofilm activity of the disinfectant P3 against *L. monocytogenes* in monoculture (Figure 1) suggested that the biofilms formed in conditions approaching the food industry environment (11°C and 1/10 diluted TSB-YE) could be less susceptible. Other authors also reported that the biofilms of *L. monocytogenes* produced at low temperatures were less susceptible to antimicrobials (Lourenço et al., 2011; Puga et al., 2016; Piercey et al., 2017). Biofilm susceptibility testing is usually performed on biofilms formed at room temperatures. Nevertheless, since most food plants have cold wet growth niches in production and storage areas, susceptibility testing should also be performed on biofilms produced at low temperatures (Lourenço et al., 2011). In addition, P3-oxonia recommended concentrations are 0.05–3%, at temperatures between 5–20°C.^1^

### Dual-Species Biofilms

The assays with dual-species biofilms proceeded at 11°C in 1/10 TSB-YE as well as in rich control medium TSB-YE. The result of cellular enumeration of *Listeria* and *Lactobacillus* at low temperatures is most probably related to the psychrotrophic and mesophilic characters of the pathogen and the LAB, respectively, since *L. monocytogenes* has the ability to grow on a wider range of temperatures than *L. plantarum*, namely at low temperatures. Nevertheless, the dominant species in the mixed biofilms is related to a variety of factors such as the combination of bacterial species in the mixture, contact surface materials, the sequence of colonization, the age of biofilms and temperature of biofilm formation, among others. For instance, the biofilm cell density of *L. monocytogenes* varied in dual-species biofilms co-cultured with *Pseudomonas*, *Staphylococcus*, or *Flavobacterium*, and the increase or decrease of the *Listeria* biofilm cell level was dependent upon its companion strain in the mixtures (Lourenço et al., 2011; Rodríguez-López et al., 2015; Wang, 2019).

Biofilms were exposed to P3 (Figure 2) and the anti-*Listeria* activity of the disinfectant was compared between mono- and dual-species biofilms with *L. plantarum*. When the concentration and time exposure of P3 was increased to 1% (v/v) for 10 min, the majority of the strains in mono- and dual-species biofilms reached the reference level of reduction (Figure 2B). Nevertheless, in the food industry, depending of the cleaning efficacy, the effective concentration of P3 could be lower than the in-use concentration and the response of the strains may dictate their eradication or persistence. Other authors have shown the influence of LAB in the susceptibility of *L. monocytogenes* biofilms to disinfectant treatment. Van der Veen and Abee (2011) reported that the mixed species biofilms of *L. monocytogenes* and *L. plantarum* showed enhanced resistance to BAC and peracetic acid. Kocot and Olszewska (2019) reported an increased resistance to quaternary ammonium compounds (QACs)-based, tertiary alkyl amine-based, and chlorine-based disinfectants. These differences may be explained by specific biofilm forming conditions since these authors incubated the biofilms at 37°C. For example, the accessory gene regulator (*agr*) locus of *L. monocytogenes* is important in the initial adhesion phase of biofilm production by *L. monocytogenes*, and the maximum expression of *agr* locus was observed at 37°C, whereas expression was the lowest at 10°C (Gandra et al., 2019).

Most studies grow biofilms at host-like temperatures or at room temperature and there are few studies available, where biofilms are grown at low temperatures approaching the real food industry conditions. Our results highlight the impact of the variability of experimental setup (i.e., microbial strains, culture media, and temperatures) on the results obtained. Consequently, special care should be taken in generalizing biofilm results since the particular environment in which they are formed may affect biofilm susceptibility. The results presented here indicate that *L. plantarum* in dual-species biofilms with *L. monocytogenes* enhanced the anti-*Listeria* activity of P3. Ramos et al. (2012) reported that a *L. plantarum* culture supernatant produced the rupture of a previously formed *P.aeruginosa* biofilm. *Lactobacillus plantarum* supernatant has been showed to contain hydrolases in its exoproteome (Boekhorst et al., 2006; O’Sullivan et al., 2009; Kleerebezem et al., 2010). It is possible that the hydrolytic enzymes found in *L. plantarum* exoproteome interfere with the integrity of the biofilm matrix. In addition, the production of hydrolases is correlated with later stages of biofilm formation (Marlow et al., 2014). Longer incubations of biofilms may be responsible for this production. This would explain that, although *L. plantarum* did not affect the number of viable *L. monocytogenes* cells in the biofilm, it did make the biofilm more susceptible to P3 exposure. Further studies must be carried out at low temperatures with mixed-species biofilms with *L. plantarum* and other LABs to clarify the effects on the impairment of pathogenic bacteria biofilms to different commercial disinfectants.

Several species of *Lactobacilli* have been considered as GRAS (*Generally Recognized as Safe)*, which identifies a microorganism or microbial derivatives as safe for use in the food industry (Cui et al., 2018; Gagliardi et al., 2018). The use of microorganisms with probiotic characteristics like LABs could improve quality, prolong food shelf-life, and control food contamination (Wei et al., 2006). Upon further research, *Lactobacilli* may be a complementary tool for controlling *L. monocytogenes* in the food industry as some authors already suggested (Castellano et al., 2004; Morandi et al., 2019).

## Conclusion

Even though there is much information available on single species biofilms, mixed species biofilms are more probable to occur in the food processing areas. In the present study, the effect of dual-species biofilms of *L. monocytogenes* with *L. plantarum* in the anti-*Listeria* activity of the hydrogen peroxide/peracetic acid based commercial disinfectant P3-Oxonia was investigated. Biofilms assays were conducted in environmental conditions similar to those present at food processing plants (stainless steel surface and low temperature) after a deficient cleaning procedure (nutrient deprivation). Results showed that although *L. monocytogenes* was the dominant species in dual-species biofilms with *L. plantarum*, it was generally more susceptible to low P3 concentration [0.5% (v/v)] than in mono-species. While dual-species were generally easier to control, biofilms produced under conditions approaching the real food industry environment may be more difficult to eradicate, resulting in the presence of persistent strains in food facilities and/or food products. These results will complement other ongoing research into the persistent *L. monocytogenes* strains used. More researches under conditions closer to the food industry environment are welcomed, for instance refrigeration temperatures, multi-species biofilms, different types of surfaces, and new disinfectants including natural active compounds.

## Data Availability Statement

The original contributions presented in the study are included in the article/Supplementary Material, further inquiries can be directed to the corresponding author.

## Author Contributions

LBo performed some of the assays and prepared the manuscript. VM executed the majority of the experiments. IB participated in the experiments. LBr projected the study, assessed, and interpreted the results. LBo and LBr wrote the manuscript. JM-S and LBo assessed and interpreted the results. All authors contributed to the article and approved the submitted version.

## Conflict of Interest

The authors declare that the research was conducted in the absence of any commercial or financial relationships that could be construed as a potential conflict of interest.

## Publisher’s Note

All claims expressed in this article are solely those of the authors and do not necessarily represent those of their affiliated organizations, or those of the publisher, the editors and the reviewers. Any product that may be evaluated in this article, or claim that may be made by its manufacturer, is not guaranteed or endorsed by the publisher.

## Footnotes

^1^ https://www.lenntech.com/Data-sheets/ECOLAB-P3-oxonia-active-L-EN.pdf
