# Supplementary figures and images for: Lactobacillus plantarum in Dual-Species Biofilms With Listeria monocytogenes Enhanced the Anti-Listeria Activity of a Commercial Disinfectant Based on Hydrogen Peroxide and Peracetic Acid
Source: Front Microbiol. 2021 Jul 30;12:631627. doi: 10.3389/fmicb.2021.631627 (PMC8363201; doi:10.3389/fmicb.2021.631627)

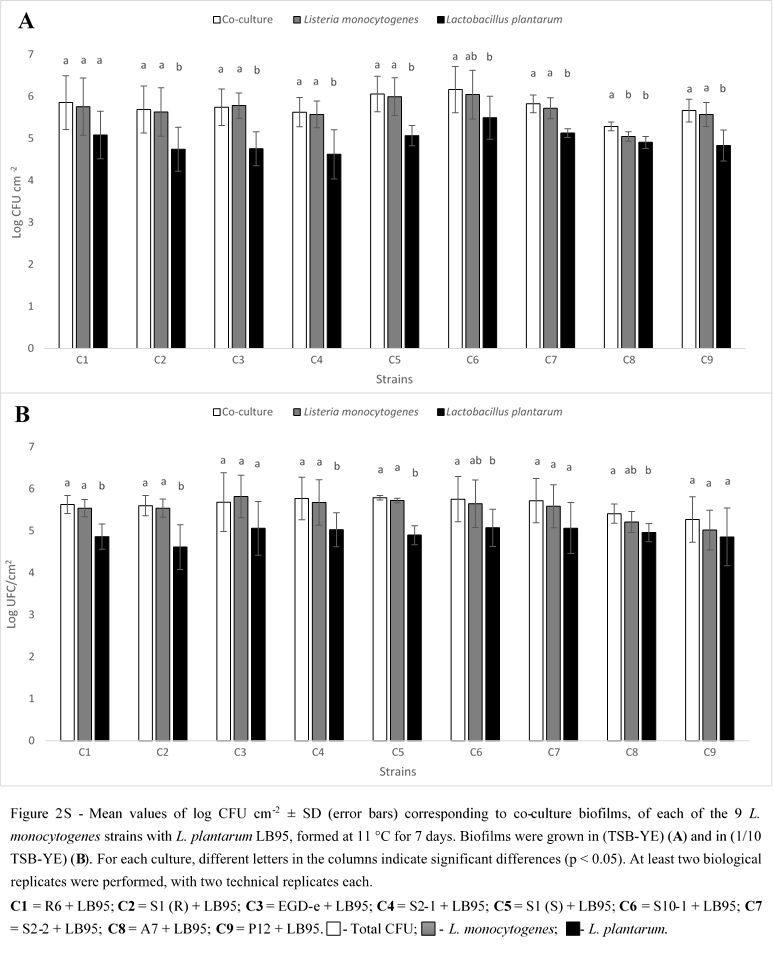

Supplement: Supplementary file 1 [file Image_1.TIF]
